# Social integration and inclusion after pre-maxilla surgical repositioning in patients with bilateral cleft palate and lip

**DOI:** 10.1016/S1808-8694(15)30493-6

**Published:** 2015-10-19

**Authors:** Kelston Ulbricht Gomes, Abrão Rapoport, João Luiz Carlini, Carlos Neutzling Lehn, Odilon Victor Porto Denardin

**Affiliations:** 1 Master's Degree Student - Graduate Program in Health Sciences - Heliopolis Hospital - São Paulo; 2 University of São Paulo Medical School (FM-USP), Technical director of the Health Department - Heliópolis Hospital; 3 PhD - Federal University of Rio de Janeiro, Maxillo-Facial Surgeon Centro de Atendimento Integral Làbio-Palatal de Curitiba, PR; 4 Federal University of São Paulo - Head of the Head and Neck Surgery and Otorhinolaryngology Department - Heliópolis Hospital - São Paulo; 5 PhD - Federal University of São Paulo; Biostatistician - Heliòpolis Hospital - Hosphel, São Paulo

**Keywords:** cleft lip, cleft palate, bone transplantation

## Abstract

Bilateral alveolar process fissure causes important functional and aesthetical limitations and thus difficulties in terms of social and family integration and interaction for these patients.

**Aim:**

(A) to assess motivations and expectations Treatment-wise and (B) to assess social inclusion and integration brought about by the surgery.

**Materials and Methods:**

retrospective observational study involving 50 participants recruited among the patients submitted to the premaxilla repositioning procedure from January of 2003 to July of 2005 at the Centro de Atendimento Integral ao Fissurado Lábio-Palatal in Curitiba (CAIF), Paraná, and an assessment questionnaire was used.

**Results:**

as far as the care protocol is concerned, results show a 90% success rate of surgeries. As to surgical and personal satisfaction rates are concerned, 76% sought treatment for personal satisfaction reasons and 86% reported having had more self-confidence after the surgery.

**Conclusions:**

Most of the patients submitted to the maxilla repositioning saw surgery as a continuation of the care previously given, with the expectation of better looks and self-confidence and, 96% of them were pleased with the results attained, which facilitated their social inclusion and re-integration.

## INTRODUCTION

Patients with bilateral fissure in their alveolar process and pre-maxillary overprojection are victims of prejudice and have relationship difficulties in their social groups. Therefore, the benefits achieved with maxillary defect reconstruction not only help with a better cosmetic appearance, but also give these children back their biological, psychological and socio-cultural identity.

Reconstruction of the maxillary defect with autogenous bone graft has benefited patients with lip-palatal cleft, especially those with unilateral alveolar clefts^1^, ^2^, ^3^, ^4^. In order to treat the premaxilla in patients with bilateral cleft there are two options: complete removal of the premaxilla (causing better lip closure^5^) and premaxillary repositioning with autogenous bone graft^6^,^7^.

Surgical treatment of patients with bilateral clefts can have severe implications since premaxilla manipulation can impair regional bone growth. There are controversies whether or not the surgical intervention in the premaxilla can be carried out before or after complete facial growth.

Besides the technical aspect, one must take into account the expectations of patient and family regarding the results of the procedure performed, since this severe facial deformity can impact the patient's inclusion and integration in his/her social environment.

After the importance of oral health was established, there has been great interest in the use of instruments to measure health-related life quality issues, with the goal of assessing the impact of oral disease or deformity on the individual's life^8^,^9^.

Thus, we tried to assess the motivations and expectations as far as treatment is concerned, as well as the individual's perception of social inclusion and integration after the procedure.

## MATERIALS AND METHODS

Our study involved a sample with 50 patients, 33 (66%) males and 17 (34.4%) females, all with bilateral transforaminal clefts; operated between January of 2003 and July of 2005 (this study was approved by our Ethics in Research Committee under protocol # 552).

The procedure was complex and the patient's social inclusion, especially in this age range, was the major benefit achieved. The present investigation aimed at studying and assessing the social consequences and the impact on the patients' quality of life, through a questionnaire which replaced the CAIF protocol. (Chart 1)

The patients were educated about the study and signed a free and informed consent form, authorizing the staff to perform the treatment proposed, as well as to disclose the results achieved. Full privacy was given to the children and their guardians (without time constraints) in order to answer the questions in the questionnaire, and the answers were submitted to a joint evaluation committee by the institution's head of pedagogy in order to assess its true social benefit. The surgical technique used is described below.

Chart 1 **Questionnaire used to assess the expectations of patients with labio-palatine clefts.**

1- Why did you look for treatment?
  a) Because I wanted to chew, speak and eat better.
  b) Because you were concerned with your physical appearance and wanted to look handsome (pretty).
  c) Because the physician or dentist said it was necessary.
  d) You looked for treatment yourself because you were concerned and embarrassed because of the problems in your mouth.
2- What did you expect to improve with surgery?
  a) To make more friends, to lighten up and to talk more at home and at school.
  b) To look better.
  c) Your school grades would improve.
  d) Would feel better with yourself.
3- Which were the changes you noticed after surgery?
  a) I looked better.
  b) Felt it became harder to say some words.
  c) Felt that life, in general, got better after correcting the problems in my mouth.
  d) Did not notice.
4- Are you pleased with treatment?
  a) Yes.
  b) No.

With the patient under general anesthesia, the rhino-pharynx was blocked by sterile gauze in order to protect the upper airways. The surgical technique used involves the injection of 2% lidocaine with 1:200.000 adrenalin in the region to be operated, in order to cause vasoconstriction and analgesia. With a 15 blade we make a vertical incision in the cleft borders, extending it posteriorly with an intrasulcus incision – 1 to 2 teeth away from the cleft. In this region we make a relief incision towards the vestibular fundus, and we raise a mucoperiosteal flap.

On the border of the premaxilla cleft we make a vertical incision and raise it only far enough to expose the remaining alveolar bone. In the palatine portion we expose the bone which supports the premaxilla. Using a reciprocating saw we make an osteotomy posteriorly to the premaxilla. Following that we incise the palatine mucosa on the palatine cleft site, keeping about 1.5cm of palatine mucosa attached to the premaxilla. The premaxilla was pushed anteriorly and, following that, the nasal mucosa is separated from the oral, and the nasal floor and is bilaterally closed with resorbable suturing wire. As soon as the nasal floor is sealed, we suture the palatine mucosa with what was left from the premaxilla palatine mucosa. The surgical guide is placed in and the premaxilla is repositioned.

In the donor area, when the mandibular symphysis is used, we make an incision in the labial mucosa, 1.5cm below the lower lip red area, cutting through mucosa and muscles, and then we push the scalpel towards the mandibular bone. After raising the periosteum with a rotating burr, we make an osteotomy with an adequate design in order to fit it to the cleft defects.

The bone graft blocks are fitted to the clefts and fixed with straight miniplates and screws. The miniplates are fitted in order to stabilize the grafts and the premaxilla to the remaining maxilla. The surgical guide, besides providing for a new premaxilla placement, it also helps supporting and fixing it, and must remain for at least two months fixed to the upper teeth arch. The graft is covered with the mucoperiosteal flap, shifted through periosteum relief incisions on the flap base. The cleft closing is done with a tension-free flap and the suture is carried out with nylon wire.

Post-op follow up was in an outpatient basis and the suture was removed in two weeks. The surgical guide was removed two months after the procedure. After such period, the patient was then referred back to his/her orthodontist in order to continue treatment.

## RESULTS

As far as treatment protocol is concerned, it involved orthodontic assessment, with the placement of brackets in the maxilla to correct the maxillary atresia and to try to better position the premaxilla. The age for surgery was established based on the position of the upper and lower canine teeth - between 8 and 12 years, and the lower ones - depending on the stage of dental eruption, graft removal from the mandibular symphysis region is done to create space for the osteotomy without damaging the teeth roots with the cutting burr (Photograph 1). Despite all of this, in most of the patients (24) the graft was removed from the mandibular symphysis region and in 26 of them we used iliac crest bone graft - for those more severely affected.

**Photograph 1:**
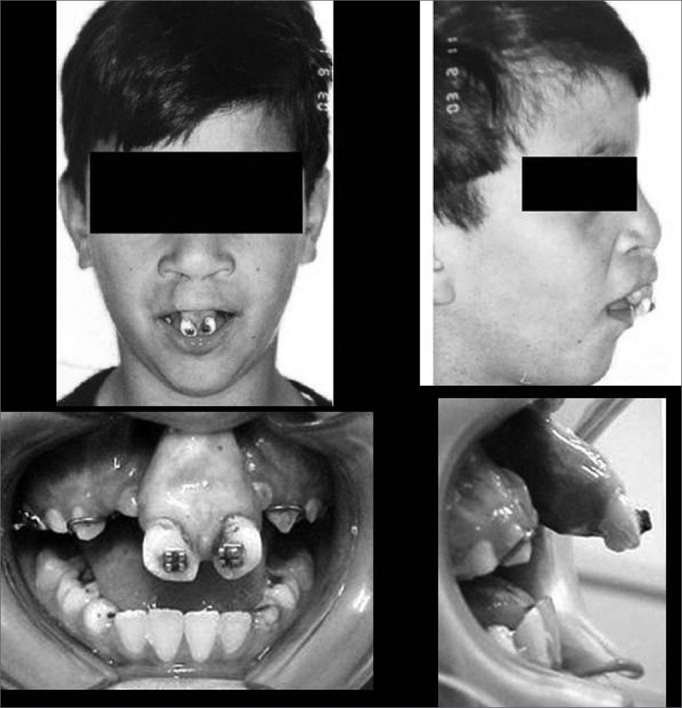
Pre-operative aspect of the labio-palatal cleft.

After the patient was considered fit for surgery, a pre-op consultation is carried out and the following tests are ordered: facial radiographs, panoramic radiograph, side-view teleradiography and upper occlusal and periapical intrabuccal radiographs of the upper incisive teeth. In the same preoperative consultation the upper and lower dental arches are molded with alginate and, by means of a simulated surgery in the gypsum casts, the premaxilla is cut and replaced in a more satisfactory position, which allows for a more satisfactory arch shape, reducing the premaxilla projection and the spaces corresponding to the cleft. At this stage an auto curing acrylic surgical guide is made in order to be used during surgery and to help position the premaxilla. The orthodontist is asked to glue the orthodontic brackets to the upper molar and incisive teeth to help fix the surgical guide using 0 steel suturing wires.

Among the 50 operated patients, 45 (90%) cases were successfully operated, there was proper graft take and the bucco-nasal clefts were closed. In the five remaining patients (10%) treatment failed or there was only partial success (one patient had bilateral graft loss; two patients had unilateral graft loss and there were two cases of premaxilla necrosis) after the first procedure. In the three cases of graft loss, the patients were submitted to a new successful intervention. In the two cases of premaxilla loss because of necrosis, new grafting procedures were carried out in order to recover the premaxilla, with satisfactory results.

The parameters used to conclude for treatment success were based on occlusal and periapical radiographs, carried out 06 months after surgery. Clinical exam showed cleft bone filling and no premaxilla movement. New periapical and occlusal radiographs were carried out 12 months after surgery in order to check for upper canine irruption and orthodontic movement of those teeth adjacent to the cleft. (Photograph 2).

**Photograph 2:**
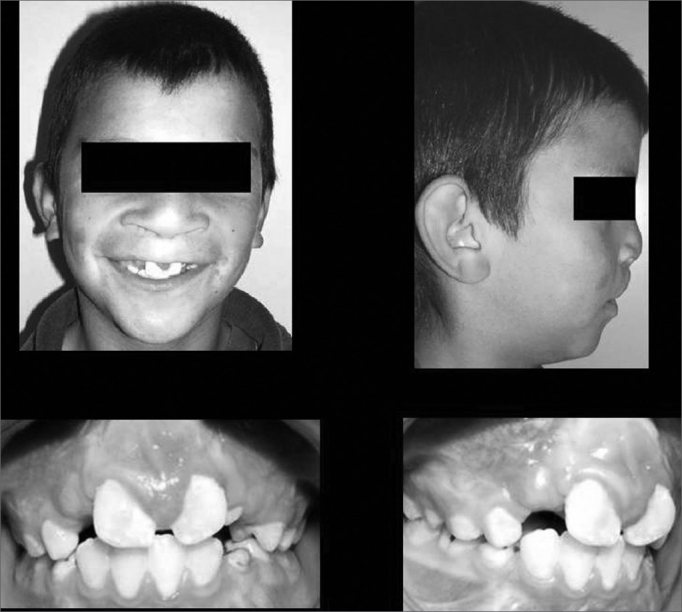
Post-operative aspect of the labio-palatal cleft.

As to the reason for surgery, 84% of the patients came because of a professional referral and only 16% came seeking improvement of aesthetics and function (Table 1).

**Table 1:** Frequency distribution of reason-to-seek-treatment variable, in the total sample.

Reason for seeking	Frequencies	
	Absolute (n)	Relative (%)
Functional improvement	3	6%
Cosmetic improvement	5	10%
Professional indication	42	84%
Your own initiative	0	0%
Total	50	100%

As to the results regarding expectations after treatment, 76% came for surgery for personal satisfaction reasons (aesthetics). 18% of the interviewed patients expected improvements in their social and affective relationships, and 6% were interested in better professional conditions (Table 2).

**Table 2:** Frequency distribution of the expectation-of-improvement-with-treatment variable, in the total sample.

Seeking reason	Frequencies	
	Absolute (n)	Relative (%)
Social/affective	9	18%
Physical/cosmetic	0	0%
Professional/school	3	6%
Personal satisfaction	38	76%
Total	50	100%

The changes seen were significant, 86% reported better self-confidence and 14% reported changes in their physical appearance (Table 3).

**Table 3:** Frequency distribution of the changes-perceived-after-treatment variable, in the total sample.

Reason for seeking	Frequencies	
	Absolute (n)	Relative (%)
Physical appearance	7	14%
Voice difficulties	0	0%
More confident	43	86%
Not noticed	0	0%
Total	50	100%

As far as result satisfaction goes, 96% were pleased (Table 4).

**Table 4:** Frequency distribution of the satisfaction-with-treatment-results variable, total sample.

Gender	Frequencies	
	Absolute (n)	Relative (%)
Satisfied	48	96%
Unsatisfied	2	4%
Total	50	100%

## DISCUSSION

Surgical intervention in patients with bilateral clefts cause great repercussion, especially regarding a procedure done to the premaxilla, considering impact on maxillary growth. Nonetheless, the age recommended for secondary bone graft is between 8 and 12 years^10^, and the damage to growth in such region may have been already caused during primary lip and/or palate closure^11^. This concept is also supported by other authors, who suggest that the pre-maxilla can be repositioned even before the end of facial growth without impairing the patient's growth outcome. They also suggest that a later class III development can be attributed to a mandibular prognathism and not a maxillary hypoplasia^12^. Other groups of researchers agree that the premaxilla osteotomy allows for a proper upper arch alignment and creates a better tilt of upper incisive teeth13. As to the type of graft used, there are many donor areas that can be used; the mandible is the best one because it bears similar embryonary origin^14^, ^15^, ^16^, ^17^.

On the other hand, many studies have analyzed the psychosocial aspects and the benefits reached with the surgical treatment of patients with bilateral cleft. They believe and assign a great importance to the maxillo-facial complex in establishing the looks of human beings, especially after analyzing those with congenital labio-palatal malformation^4^,^18^. Children with labio-palatine clefts at school age usually face a very negative social experience regarding the reaction of children and other people when looking at them. The initial difficulties tend to minimize with time and rehabilitation, which must start as soon as possible^9^.

One aspect to be stressed is the repercussion of the treatment proposed bringing about a raise in the self-esteem of these patients. Satisfaction leads the individual to feel confident, useful and necessary to the world. Dissatisfaction causes a feeling of inferiority, weakness and powerlessness in their lives or varied disease processes^19^. A person with chronic low self-esteem may develop health problems^20^,^21^.

In the present investigation, we associated the improvements reached in home life and school performance with an increase in the self-esteem of these children. This correlation could be based on the fact that it is at school that children have to face new relationships and peer judgment, and their facial looks play a very important role in this judgement^9^. Adding to this idea, there are authors who advocate the existence of a personal identity that others assign among themselves based on their physical appearance. The person the individual becomes is a historical construction which includes self-representation and the representation that others make of them. The body gathers a number of fears and fantasies which establish the degree of satisfaction the individual has with him/herself^22^,^23^. The concept of self-esteem has been studied and considered an important mental health indicator^24^, ^25^, ^26^. Criticism, in general, establishes the need to employ accurate tools to assess the self-esteem needs of each individual, especially in children. For this reason, it is worth to perform this study assessing the levels of self-esteem in children with bilateral clefts of their alveolar processes.

The analysis of life quality assessment questionnaires in children shows the lack of consensus regarding the use of an assessment tool. Whenever the child is skilled at providing reliable and valid data, its direct application in these patients is the ideal strategy because if matches the very definition of quality of life which emphasizes the patient's subjectiveness^27^.

An assessment tool like this one, employed by means of an interview may be more expensive than the questionnaire employed. For very young children or those severely impaired, those in charge can provide information which could not be gathered otherwise. The perspective of the guardians is to assess the impact of health on children and decide whether or not this child should be treated based on the determining variables of family dynamics, which is an integral part of the child's quality of life^28^.

There are numerous disadvantages in using parents as respondents. First, a report by a representative does not match a concept of quality of life, which is defined according to the patient's subjective opinion^28^. Second, the report from fathers and mothers may not match^29^,^30^, it is recommended that the parents be assessed and then the information be crossed, in order to find possible errors^28^. Third, the report of guardians on the impact of the disease on their children is based on knowledge about how there are affected themselves. Finally, it is not totally clear whether the parents would be the best adults to answer the questionnaire^29^, since some children can spend more time with teachers and care givers or other family members than parents, and thus another adult would have better knowledge regarding the social and psychological functioning of the child^28^. Because of this lack of consensus, some researchers have suggested the need to obtain information from guardians and children^30^. This approach may provide more complete information of how either the disease or the treatment impacts the lives of children and their families^28^. Nonetheless, authors^31^ state that it is clear that we are yet very short of a uniform and universal conception of quality of life in childhood, and also regarding the means to assess this concept adapted to the pediatric universe. It is a priority to have it very clear the need to instate definitions which translate the interests of children and adolescents, and not the adults who assess them, and that assessment methods be installed to capture the individuals' perceptions to be assessed, and not the expectations and perceptions of the caregiver, being him a parent or a health-care professional.

Thus, the self-assessment of oral health and global satisfaction with life gives children and their guardians the opportunity to express their own personal understanding of reality and health. The results found in the present study regarded the sample studied and cannot be generalized for the entire population of people with bilateral clefts from the Centro de Atendimento Integral ao Fissurado Lábio-Palatal, and the size of this sample is the very limiting factor of this study. After analyzing the data seen on the tables, one can notice that although there was a concern with the cosmetic improvement, the acceptance of surgery by most of the patients (84%) happened because of this institution's suggestion because, as described before, patients who received a long standing rehabilitative treatment are quickly adapted to medical and technical procedures at the hospital, which may not be positive since patients may not respond to treatment with the proper autonomy^32^.

The value assigned to physical appearance contributed to 10% of the patients studied seeking surgery; this can be impacted by the fact that ours is a western country geared to beauty^33^. Although functional problems are the major argument used to justify surgical intervention, the patients see significant changes in their social relations, because with the new facial and speech improvement, they feel safer to be accepted by the group to which they belong^22^,^24^.

As to the changes, surgery causes a feeling of liberation to the patient, one of being ready to socialize and start interpersonal relations. Surgery constitutes a concrete consent to the externalization of repressed desires and wishes^25^.

## CONCLUSIONS

Treated patients agree that the results caused cosmetic harmony, besides changing interpersonal relations and increasing self-esteem; better social integration and greater cooperation regarding parallel treatment and recovery^20^.

## References

[1] Boyne PJ (1970). Autogenous cancellous bone and marrow transplants. Clin Orthop..

[2] Boyne PJ, Sands NR (1972). Secondary bone grafting of residual alveolar and palatal clefts. J Oral Surg..

[3] Spina V (1973). A proposed modification for the classification of cleft lip and palate. Cleft Palate Cranio-Fac J..

[4] Andrade D, Angerami ELS (2001). A autoestima em adolescentes com e sem fissuras de lábio e/ou de palato. Rev Latinoam Enferm..

[5] Aburezq H, Daskalogiannakis J, Forrest C (2006). Management of the prominent premaxilla in bilateral cleft lip and palate. Cleft Palate Cranio-fac J..

[6] Bergland O, Semb G, Abyholm F (1986). Elimination of the Residual Alveolar Cleft by Secondary Bone Grafting and Subsequent Orthodontic Treatment. Cleft Palate Cranio-fac J..

[7] Ferreira RA (1997). Odontologia: essencial para a qualidade de vida. Rev Assoc Paul Cirur Dent..

[8] Bortoli D, Locatelli FA, Fadel CB, Baldani MH (2003). Associação entre Percepção de Saúde Bucal e Indicadores Clínicos e Subjetivos: Estudo em Adultos de um Grupo de Educação Continuada da Terceira Idade. Publ. UEPG Ci. Biol Saúde..

[9] Amaral VLAR (1986). Vivendo com uma face atípica: influência da deformidade facial e auto e hetero conceitos e na realização acadêmica de crianças de 6 a 12 anos.

[10] Enemark H (1988). Comparative study of secondary and late secondary bone-grafting patients with residual cleft defects. Short-term evaluation. Int J Oral Maxillofac Surg..

[11] Freihofer HPM, Jagtman AK (1989). Early Secondary Osteoplastic Closure of the Residual Alveolar Cleft in Combination with Orthodontic Treatment. J CranioMaxillof Surg..

[12] Padwa B, Sonis A, Bagheri S, Mulliken J (1999). Children with repaired bilateral cleft lip/palate: effect of age at premaxillary osteotomy on facial growth. J Plast Reconstr Surg..

[14] Brouns J, Egyedi P (1980). Osteotomy of the premaxilla. J Maxillofac Surg..

[15] Banks P (1983). The surgical anatomy of secondary cleft lip and palate deformity and its significance in reconstruction. Br J Oral Surg..

[16] Scott JK, Webb RM, Flood TR (2007). Premaxillary Osteotomy and Guided Tissue Regeneration in Secondary Bone Grafting in Children With Bilateral Cleft Lip and Palate. Cleft Palate Cranio-fac J..

[17] Koole R, Bosker H, Dussen NVD (1989). Late Secondary Autogenous Bone Grafting in Cleft Patients Comparing Mandibular (Ectomesenchymal) and Iliac Crest (Mesenchymal) Grafts. J Craniomaxillof Surg..

[18] Leonard BJ, Brust JD, Abrahams G, Sielaff B (1991). Self-concept of children and adolescents with cleft lip and/or palate. Cleft Palate Craniofac J..

[19] Maslow AH (1970). Motivation and personality.

[20] Campos RG (2001). Eu tenho a força. Rev Viver.

[21] Carvalho LEP. Avaliação do nível de satisfação, capacidade, eficiência e performances mastigatórias em pacientes reabilitados com próteses fixas totais inferiores sobre implantes, sob carga imediata. Dissertação (Mestrado em Implantodontia)-Universidade do Sagrado Coração, Bauru. 100f, 2002.

[22] Alves MCR (1985). Aspectos psicológicos das intervenções cirúrgicas na área odontológica. Odontol Mod..

[23] Romero E (1998). As dimensões da vida humana: existência e experiência.

[24] Fadiman J, Frager R (1979). Teoria da personalidade.

[25] Wolf SMR (1998). O significado psicológico da perda dos dentes em sujeitos adultos. Rev Assoc Paul Cir Dent..

[26] Cano MAT, Ferriani MGC, Alves AC, Nakata CY (1998). A produção do conhecimento sobre adolescência na enfermagem: período de 1983 a 1996. Rev Latinoam Enferm..

[27] Eiser C, Mohay H, Morse R (2000). The measurement of quality of life in young children. Child: Care, Health and Develop..

[28] Matza LS, Swensen AR, Flood EM, Secnik K, Leidy NK (2004). Assessment of health-related quality of life in children: a review of conceptual, methodological, an regulatory issues. Value Health..

[29] Landgraf JM, Abetz LN, Spilker B (1996). Quality of life and Pharmacoeconomics in clinical trials.

[30] Eiser C, Morse R (2001). Can parents rate their childs health-related quality of life? Results of a systematic review. Qual Life Res..

[31] Assumpção FB (2000). Escala de avaliação de qualidade de vida (AUQIE Autoquestionnaire Qualité de Vie Enfant Imagé): validade e confiabilidade de uma escala para qualidade de vida em crianças de 4 a 12 anos. Arq Neuropsiquiatr..

[32] Veronez FS, Tavano LDA (2005). Modificações psicossociais observadas póscirurgia ortognática em pacientes com e sem fissuras labiopalatinas. Arq Ciênc Saúde..

[33] Garvill J, Garvill H, Kahnberg KE, Lundgren S (1992). Psychological factors in orthognathic surgery. J Craniomaxillofac Surg..

